# Sociability: Comparing the Effect of Chlorpyrifos with Valproic Acid

**DOI:** 10.1007/s10803-024-06263-z

**Published:** 2024-03-11

**Authors:** Miguel Morales-Navas, Cristian Perez-Fernandez, Sergio Castaño-Castaño, Ainhoa Sánchez-Gil, María Teresa Colomina, Xavier Leinekugel, Fernando Sánchez-Santed

**Affiliations:** 1 Department of Psychology and Health Research Center (CEINSA), University of Almería, Ctra. Sacramento, s/n, 04120 Almería, Spain; 2 Departamento de Psicobiología, Facultad de Psicología, Universidad de Oviedo, Plaza de Feijoo, 33003 Oviedo, Asturias Spain; 3 Department of Psychology and Research Center for Behavior Assessment (CRAMC), Universitat Rovira i Virgili, C/Carretera de Valls, s/n, 43007 Tarragona, Spain; 4 Institut de Neurobiologie de la Mediterranée (INMED), INSERM UMR1249, Aix-Marseille University, Parc Scientifique de Luminy BP.13, CEDEX 09, 13273 Marseille, France

**Keywords:** Autism, Sociability, Chlorpyrifos, Pesticides, Valproic acid

## Abstract

In recent years, exposures to organophosphate pesticide have been highlighted as a possible cause or aggravating factor of autism spectrum disorder (ASD). The present study examined if Wistar rats prenatally exposed to chlorpyrifos (CPF) at a dose of 1 mg/kg in GD 12.5–15.5 could express similar behaviors to those exposed to valproic acid (VPA, 400 mg/kg) during the same administration window, which is an accepted animal model of autism. The 3-chambered test was employed to evaluate sociability and reaction to social novelty in two experiments, the first in adolescence and the second in adulthood. The results obtained in this study show that animals prenatally treated with CPF or VPA show a similar behavioral phenotype compared to the control group (CNT). In adolescence, the CPF animals showed a negative index in the reaction to social novelty, followed closely by the VPA, while both experimental groups showed a recovery in this aspect during adulthood. This study therefore provides evidence to suggest that prenatal exposure to CPF in rats could have similar effects on certain components of sociability to those seen in autistic models.

**Supplementary Information:**

The online version contains supplementary material available at 10.1007/s10803-024-06263-z.

## Introduction

Autism spectrum disorder (ASD) is a neurodevelopmental disorder characterized by repetitive behaviors in addition to cognitive and social skills deficits—both verbal and non-verbal (Gyawali & Patra, 2019). The worldwide prevalence of ASD is estimated at around 1%, although this percentage is considered to be higher in developed countries. Furthermore, it is also known that this disorder seems to affect boys more than girls (Lai et al., 2014; Lord et al., 2020). The causes of this disorder are still unknown. However, in the current literature, there is a general consensus that the etiology of this disorder is multifactorial, including genetic or environmental factors (Schaaf et al., 2020). Regarding these lasts, exposure to pesticides, and more concretely, prenatal exposure to Chlorpyrifos (CPF), an organophosphate (OPs), has been considered as a possible contributing element in the development of autism (Persico & Merelli, 2014).

The main mechanism of action of CPF is the inhibition of acetylcholinesterase (AChE), which causes overstimulation of cholinergic receptors, leading to collapses within the nervous system (US Department of Health & Human Services, 1997). During prenatal development, CPF exposure at toxic doses ranges from disruption of cell proliferation, differentiation, and apoptosis (Raszewski et al., 2015) to impaired dendritic maturation (Howard et al., 2005).

In humans, according to a study conducted in California, the risk of presenting ASD was twice as high for those children born from mothers who, during gestation, were exposed to pesticides (among them CPF) due to their proximity to crop fields (Shelton et al., 2014). This data is supported by other studies that showed abnormalities in the brains of children who had been prenatally exposed to CPF, finding that these children also obtained lower scores on intelligence tests. In addition, such a link to pesticide exposure has also been reported in autistic children (Gunier et al., 2017; Rauh et al., 2012).

In rodent research in which the control of intervening variables is more precise, it has been shown how prenatal exposure to CPF, at doses that do not cause AChE inhibition, results in impairments similar to those observed in ASD. These impairments include decreased social behaviors (Lan et al., 2017) reduction in the number and length of ultrasonic vocalizations emitted (Venerosi et al., 2009), and even hyperactive behaviors (Grabovska & Salyha, 2015), something that has been associated (due to high comorbidity rates) with ASD (Sokolova et al., 2017).

However, despite these data, it is not entirely clear whether prenatal exposure to CPF is related to ASD (Biosca-Brull et al., 2021; Williams & Desesso, 2014). Thus, to go a step further in this regard, it is useful to employ an accepted autistic animal model, such as valproic acid (VPA; Ergaz et al., 2016; Mabunga et al., 2015). This model of ASD has been confirmed by studies carried out using rats and mice since, after prenatal exposure to the drug, they show characteristics very similar to those expected in individuals with ASD (Chaliha et al., 2020; Mabunga et al., 2015). These features include a reduction in the production of ultrasonic calls in isolated pups (Cezar et al., 2018; Dai et al., 2018; Morales-Navas et al., 2020), increased levels of anxiety and stereotypical behaviors (Bronzuoli et al., 2018; Servadio et al., 2015) and deficits in social interactions in the 3-chambered test (Bambini-Junior et al., 2014; Bronzuoli et al., 2018; Kim et al., 2014).

In addition, there are also significant similarities between the neurobiological effects of prenatal exposure to VPA and CPF. For example, GABAergic alterations have been observed in animals exposed perinatally to subclinical doses of CPF, which do not irreversibly inhibit AChE (Perez-Fernandez et al., 2020b), and in animals treated prenatally with VPA (Hou et al., 2018; Wei et al., 2016). In fact, metabolomics analyses of prenatal administration of CPF and VPA pointed out an alteration in the cerebral levels of glutamine, GABA, and choline (Abreu et al., 2021). This is very important if we consider that the GABA dysfunctions appear to play in the brain's excitatory/inhibitory electrophysiological balances and also the suggested role of the alterations in the cholinergic system in the etiology of ASD (Gogolla et al., 2009; Wang et al., 2015; Ford & Crewther, 2016; Hou et al., 2018).

Given the above considerations, the present study set out to conduct, for the first time, a direct comparison between the social interaction of rats prenatally exposed to subclinical doses of CPF and those exposed to VPA within the same period of fetal development. To this end, two experiments were conducted to evaluate social behavior using the 3-chambered test in different groups of rats (from the same litters): one in adolescence and the other in adulthood.

## Methods

### Experimental Animals

The mothers were twenty-five 3-month-old pregnant Wistar rats (Janvier Labs; Le Genest-Saint-Isle, France) housed individually in transparent polycarbonate cages (50 × 15 × 24 cm) in our Facility. For 6 days, the rats were acclimatized to their new environment, regulated at a temperature of 22 ± 2 °C and humidity at 50 ± 10%. The light/dark cycle was reversed, with lights on from 19:00 to 07:00 h. All rats gave birth on the expected day, postnatal day 0 (PND0).

On the following day (PND1), all pups were separated from their mothers to be randomly distributed among them, keeping a ratio of five females and five males per mother to minimize differences in rearing. Making a total of 10 pups per mother, among which a balanced number of 3 ± 1 pups per experimental group was maintained. The mothers had continuous free access to water and food. Furthermore, the offspring were weighed regularly to monitor possible signs of intoxication. We started with this last routine at PND10 as we wanted to prevent extreme maternal reactions that could affect the offspring. This study is part of the project PSI2017-86847-C2-1-R and was conducted following the Royal Decree 53/2013 and the European Community Directive (2010/63/EU) for Animal Research and approved by the Animal Research Committee of the University of Almeria (29/05/2020/067).

### Administration Protocol

On Gestation day (GD) 11, the mothers were randomly assigned to one of the following three experimental groups: control group (CNT), chlorpyrifos group (CPF), and valproic acid group (VPA). Then, at GD 12.5, all pregnant mothers started treatment according to the following protocol:

*CNT* (*n* = *8*) a subcutaneous injection of 1 mL/kg dimethyl sulfoxide (DMSO) for 4 days (vehicle).

*CPF* (*n* = *8*) a subcutaneous injection of 1 mg/kg of CPF [O, O-diethyl O-3, 5, 6-trichloropyridin-2-yl phosphorothioate (Pestanal, Sigma Aldrich)] dissolved in DMSO (100 mg/mL) for 4 days.

*VPA* (*n* = *9*) one subcutaneous injection of 400 mg/kg of VPA (to avoid possible maternal death Vorhees, 1987), dissolved in 0.9% saline at a concentration of 250 mg/mL, and three daily subcutaneous injections with only saline solution.

Pregnant mothers were weighed daily to ensure that the correct doses were administered.

The schedule of administrations was completed at GD 15.5 when all mothers had received four subcutaneous injections each (Table 1).

**Table 1:** Schedule of doses

Group	12.5 GD	13.5 GD	14.5 GD	15.5 GD
Control	DMSO	DMSO	DMSO	DMSO
CPF	CPF	CPF	CPF	CPF
VPA	VPA	Saline	Saline	Saline

### Sociability Test

The apparatus consists of a rectangular box (96 × 105 cm) based on that designed by Crawley (2004, which has three different chambers (96 × 35 cm): one in the center and two on both sides. The chambers are separated by glass to allow the animal to see from the exit chamber (the central one) what is in the other two. The side chambers were virtually divided into:

- *Total zone* comprising the entire chamber.
- *Contact zone* comprising the perimeter of the cages where the conspecifics of the last two sessions of the test were to be placed.

All the animals that passed the test explored the apparatus the day before for 10 min to avoid novel behavior that could interfere with the measurements. The experiments were carried out between 8:00 and 14:00 h. The test consisted of three consecutive phases, all lasting 10 min. After each animal had been tested, the apparatus was cleaned with a 70% alcohol solution.

- *Session 1*: *acclimatization of the animal*. During this session, the animal was allowed to explore only and exclusively the central chamber, with access to the two other chambers closed. This session was used to measure the locomotor activity of the subjects. After 10 min, the subjects were momentarily removed from the apparatus before moving on to the next phase.
- *Session 2*: *sociability*. In this session, the animal's social behavior was measured by placing a congener in one of the lateral chambers. This congener (Stranger 1) was enclosed in a metal cage that allowed, if necessary, contact between the subjects. In this phase, the time spent in each chamber and the contact time with the congener were measured, as well as the Sociability Index (calculated with the time spent in each chamber) and the Sociability Contact Index (calculated with the time spent inside the perimeters near the cages where the congeners were placed). In addition, the latencies of the first entry to each chamber were also measured, as well as the number of entries to the contact zone. Before the next test, the subject was momentarily removed from the apparatus.
- *Session 3*: *reaction to social novelty*. During this phase, the response to social novelty shown by the subjects was evaluated. Thus, in this phase, in addition to the now known congener (Familiar), a new individual (Stranger 2) was placed in the cage of the other chamber. The time spent in each chamber and inside of the contact zone with each individual was then measured, as well as the Social Novelty Reaction Index (calculated with the time in each chamber) and the Social Novelty Contact Reaction (calculated with the time spent within the perimeters near the cages where the conspecifics were placed). In addition, the latencies of the first entry to each chamber were measured again, as well as the number of entries to the contact zone.

The animals were tracked using Ethovision 3.1, with an overhead camera as input. The light was subtly arranged so as not to disturb the animals.

The groups of animals that were part of the two experiments were:

- *In adolescence* [*postnatal day 37–46*; (*PND 37–46*)] 20 CNT (10 females and 10 males), 21 CPF (11 females and 10 males), and 20 VPA (10 females and 10 males).
- *In adulthood* (*PND 180–189*) 20 CNT (10 females and 10 males), 21 CPF (10 females and 11 males), and 20 VPA (10 females and 10 males).

### Statistical Analysis

The statistical analyses used for the 3-room test were ANOVA for parametric data and the Holm–Sidak multiple comparisons test for post hoc data, while the non-parametric data were analyzed using Friedman's statistic and the Kruskal–Wallis analysis of variance. For multiple comparisons, within these non-parametric analyses, Dunn's multiple comparisons were used. Finally, when the analyses consisted of only two groups, the Student's t-test was used for parametric analyses and the Wilcoxon test for non-parametric analyses.

The following formulas were used to measure the indices of sociability and reaction to social novelty (Bambini-Junior et al., 2014; Kim et al., 2014):

*Phase 2—Sociability*

$$Sociability\, Index= \frac{time\, with\, Stranger\, 1- time \,in \,empty\, compartment}{ time\, with \,Stranger\, 1+time\, in\, empty\, compartment},$$

$$Sociability\, Index{\text{-}}Contact= \frac{time\, in\, the\, perimeter\, closest \,to\, Stranger\, 1- time \,in\, the\, perimeter\, closest\, to\, the\, empty \,compartment}{ time\, in\, the\, perimeter \,closest\, to\, Stranger\, 1+time \,in\, the\, perimeter \,closest\, to\, the\, empty \,compartment}.$$

Phase 3—Reaction to Social Novelty

$$Social\, Novelty \,Index= \frac{time\, with \,Stranger\, 2-time\, with\, family\, member}{ time\, with\, Stranger\, 2+time\, with\, a \,family\, member},$$

$$Social \,Novelty\, Index{\text{-}}Contact= \frac{time \,in \,the\, perimeter\, closest\, to\, Stranger\, 2- time\, in\, the\, perimeter\, closest\, to\, the\, family \,member}{ time \,in \,the\, perimeter\, closest \,to \,Stranger\, 2+ time\, in\, the\, perimeter\, closest\, to \,the\, family\, member}.$$

Based on the assessments of Bambini-Junior et al. (2014), those subjects who did not spend time with the previous congener in Phase 2 and those who did not visit the three chambers in Phase 3 were eliminated.

All analyses were conducted using GraphPad Prism 8.0 software (San Diego, CA, USA).

## Results

### Sociability in Adolescents

#### Phase 1

**Fig. 1:**
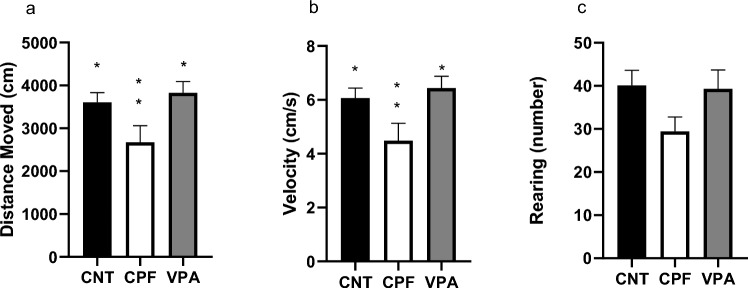
Results of locomotor behavior of the 3-chambered test in adolescents—Phase 1 [n = 61; CNT = 20 (10 females and 10 males), CPF = 21 (11 females and 10 males), VPA = 20 (10 females and 10 males)]: in Total Distance, we found significant differences between the CPF group and the other two groups; with the CPF group covering the least distance (**a**). Significant differences were also found for speed showing that the CPF group moved slower than the subjects of the other groups (**b**). No significant differences were found for rearing behavior (**c**). *Significant difference with one group. **Significant differences between the two groups

In this phase, significant differences were obtained between treatment groups in the Total Distance variable (Kruskal–Wallis analysis of variance, p = 0.01); with the CPF group covering the least distance and moving slower compared to the CNT and VPA groups (Fig. 1a, b). No significant differences were obtained for rearing behavior (Fig. 1c).

#### Phase 2: Sociability

**Fig. 2:**
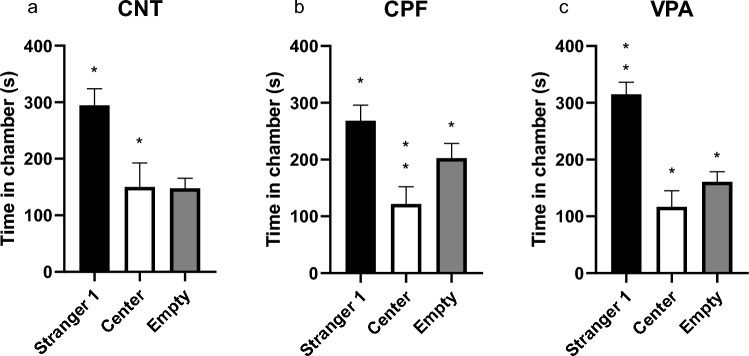
Time spent in each chamber in the adolescent 3-chambered test—Phase 2 [n = 61; CNT = 20 (10 females and 10 males), CPF = 21 (11 females and 10 males), VPA = 20 (10 females and 10 males)]: both CNTs and VPAs clearly spent more time with Stranger 1 than in the central chamber (CNT) and empty chamber (VPA) (**a** and **c**). The CPFs, although showing the same significant difference in time spent with Stranger 1 and the central chamber, also spent significantly more time in the empty chamber than the central chamber (**b**)

Concerning the time spent in each chamber according to treatment, we found that, in isolation, both the CNT group (Friedman statistic, p = 0.000) and the VPA group (Friedman statistic, p = 0.000) spent significantly more time with Stranger 1 (Fig. 2a and c, respectively). However, for the CPF group there was no clear difference between time spent with Stranger 1 or in the empty chamber (Fig. 2b).

**Fig. 3:**
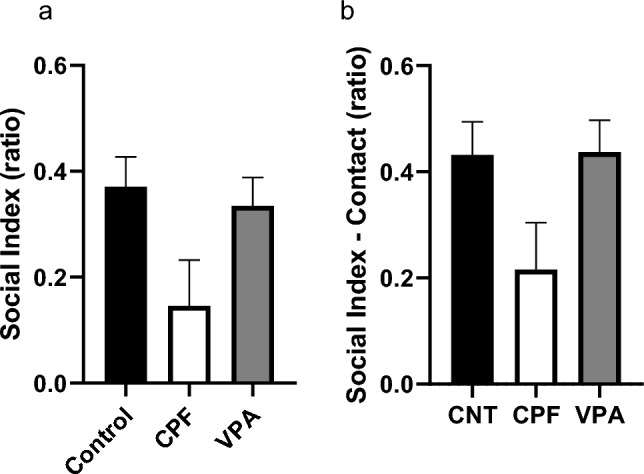
Sociability indexes for the 3-chambered test in adolescents—Phase 2 [n = 61; CNT = 20 (10 females and 10 males), CPF = 21 (11 females and 10 males), VPA = 20 (10 females and 10 males)]: despite finding a trend in the CPFs, there are no significant differences in either the Total or Contact index (**a** and **b**, respectively)

To analyze the sociability between the experimental groups, we calculated the Sociability Index since this is used to compare the different treatments. However, as the data show, no significant differences were found in the Sociability Index (SI) or the Sociability Index-Contact (SI-Contact) (Fig. 3a and b, respectively), even though the CPFs tend to be less sociable.

At this stage, although there were intrinsic differences in the CNTs in terms of the number of entries in contact (Fig. 11) and the latency of their first entry (Fig. 12), there was no difference between the groups, and these figures are therefore included in the Appendix.

#### Phase 3: Reaction to Social Novelty

**Fig. 4:**
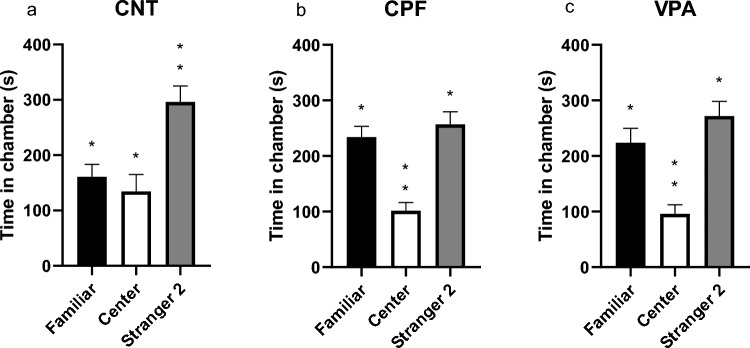
Time in each room in the Social Novelty Reaction phase in adolescents in the 3 chambered test—Phase 3 [n = 54; CNT = 16 (10 females and 6 males), CPF = 19 (10 females and 9 males), VPA = 19 (9 females and 10 males)]: CNTs show a significant difference between time spent with Stranger 2 and the other two chambers (**a**). In contrast, CPFs and VPAs show significant differences in time spent with both Familiar and Stranger 2, as opposed to time spent in the central chamber (**b** and **c**, respectively)

In this phase, there were significant differences in the total time spent in each chamber according to the treatments. For example, CNTs showed a significant preference for spending time with Stranger 2 as opposed to the central chamber or the one where the Familiar animal was located (Friedman statistic, p = 0.000, Fig. 4a). However, both CPFs (Friedman statistic, p < 0.000) and VPAs (Friedman statistic, p < 0.000), we did not show this preference for the new stranger but instead appeared to show indifference between spending time with the Familiar animal or Stranger 2 (Fig. 4b, c).

**Fig. 5:**
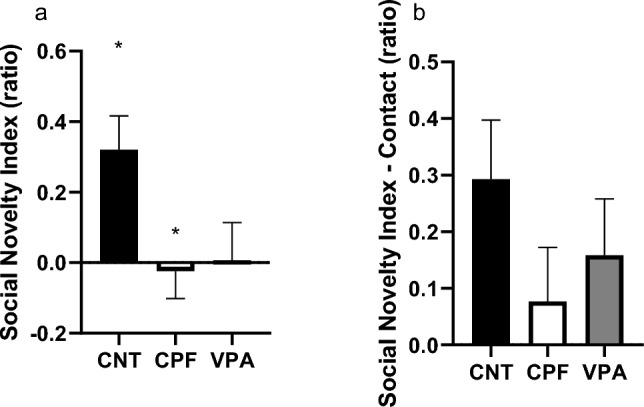
Indexes of Reaction to Social Novelty in adolescents in the 3-chambered test—Phase 3 [n = 54; CNT = 16 (10 females and 6 males), CPF = 19 (10 females and 9 males), VPA = 19 (9 females and 10 males)]: in the SNI, we can observe how the CPFs differ significantly from the CNT, the latter being much less reactive to the new stranger. In the SNI-Contact, although the basic shape of the graph is the same, the significant differences disappear

Again, to check whether these differences are significant in the experimental groups, we calculated the Social Novelty Index (SNI) and, as we can see in Fig. 5a, there are significant differences between the CNTs and the CPFs (Kruskal–Wallis statistic, p = 0.044), with the CPFs being the least reactive to social novelty. For the Social Novelty Index-Contact (SNI-Contact), we found no significant differences between the experimental groups, although the index shows a “fingerprint: of the total result (Fig. 5b).

During this phase, no significant differences were obtained in first entry latency, while for the number of contact zone entries, only the CNTs showed an intra-group difference (Figs. 13 and 14, respectively).

### Sociability in Adults

#### Phase 1

**Fig. 6:**
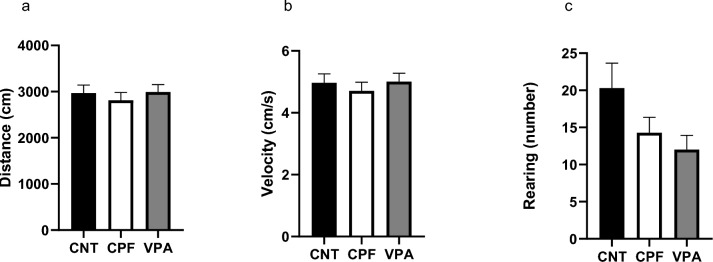
Results of locomotor aspects of the 3-chambered test in adults—Phase 1 [n = 61; CNT = 20 (10 females and 10 males), CPF = 21 (10 females and 11 males), VPA = 20 (10 females and 10 males)]: none of the groups presented significant differences in any of the variables: Total Distance (**a**), Speed (**b**), and Rearing (**c**)

No significant differences were found in any of the variables studied (Fig. 6).

#### Phase 2: Sociability

**Fig. 7:**
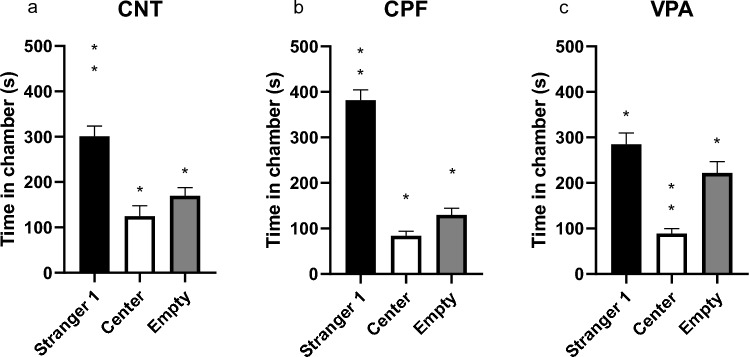
Time spent in each chamber in the adult 3-chambered test—Phase 2 [n = 61; CNT = 20 (10 females and 10 males), CPF = 21 (10 females and 11 males), VPA = 20 (10 females and 10 males)]: CNTs and CPFs spent more time in the Stranger 1 chamber than in the other two central chambers (**a** and **b**, respectively). VPAs, in contrast, spent more time with Stranger 1 or the empty chamber than in the central chamber (**c**)

In the time spent by subjects in the empty and central chambers and where Stranger 1 was located, we found that both CNTs (Friedman statistic, p < 0.000; Fig. 7a) and CPFs (Friedman statistic, p < 0.000; Fig. 7b) spent significantly more time with Stranger 1 than in the central or empty chambers. In contrast, while VPAs spent more time in the Stranger 1 chamber than the central chamber, this did not differ from time spent in the empty chamber. The only differences were in time spent in either of these two chambers compared to the central chamber (Friedman statistic, p < 0.000; Fig. 7c).

**Fig. 8:**
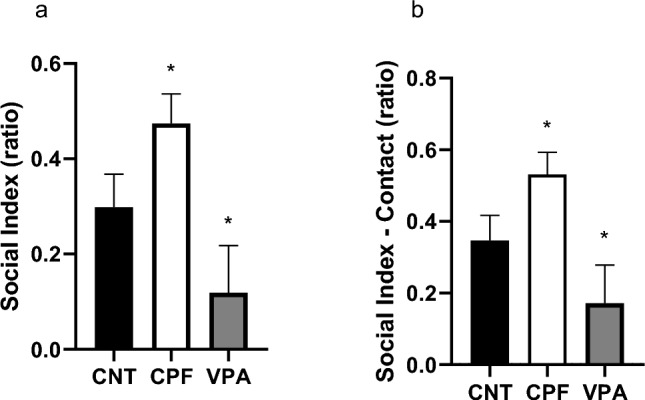
Sociability indexes in the adult 3-chambered test—Phase 2 [n = 61; CNT = 20 (10 females and 10 males), CPF = 21 (10 females and 11 males), VPA = 20 (10 females and 10 males)]: in both the Sociability Index (which considers the whole space) and the SI-Contact, CPFs are significantly more sociable than VPAs (**a** and **b**)

Group comparisons of the SI and SI-Contact revealed a significant difference between the CPFs and the VPAs, the former being more sociable than the latter, as measured by both the SI (Kruskal–Wallis statistic, p = 0.028; Fig. 8a) and the SIC (Kruskal–Wallis statistic, p = 0.020; Fig. 8b).

No significant differences were found for the latency of the first entry (Fig. 15), while for the number of contact entries, all groups showed more entries into the Stranger 1 chamber.

#### Phase 3: Reaction to Social Novelty

**Fig. 9:**
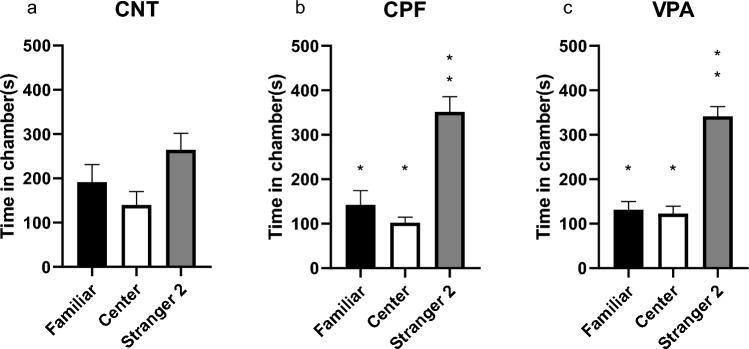
Time spent in each chamber in the adult 3-chambered test—Phase 3 [n = 50; CNT = 15 (9 females and 6 males), CPF = 18 (9 females and 9 males), VPA = 17 (9 females and 8 males)]: CNTs showed no preference for being with the new stranger or either chamber (**a**). CPFs and VPAs show a clear preference for being with Stranger 2 rather than in the other two chambers (**b** and **c**)

Here, unlike the previous phase, the within-group analysis revealed that the CNTs showed no significant differences in time spent with Stranger 2 or the familiar animal or stranger located in the central chamber (Fig. 9a). However, both CPFs (Friedman statistic, p = 0.000) and VPAs [F(1810, 28.96) = 27.18; p < 0.000] show significant differences in time spent with Stranger 2 and the other chambers (Fig. 9b, c).

**Fig. 10:**
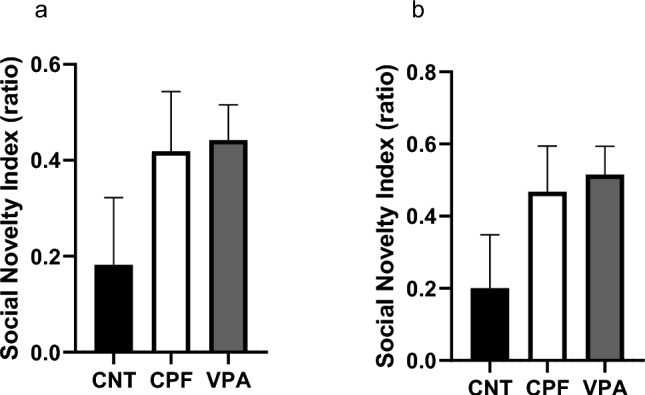
Indexes of reaction to social novelty—Phase 3 [n = 50; CNT = 15 (9 females and 6 males), CPF = 18 (9 females and 9 males), VPA = 17 (9 females and 8 males)]: no significant group differences were found for the SNI (**a**) or SNI-Contact (**b**)

Regarding SNI and SNI-Contact, no significant differences were found between the experimental groups (Fig. 10a, b).

We found no significant differences in the latency of the first entry in this phase (Fig. 15). However, in the number of contact entries, CPFs and VPAs made significantly more entries in contact with Stranger 2 than with the Familiar animal (Fig. 16).

## Discussion

In this study, we aimed to explore potential similarities between animals prenatally exposed to VPA and CPF, using, in this latter, subclinical doses routinely found in fetal brains (Gu et al., 2020) in an administration window equivalent to the first trimester of human fetal development. This time window is frequently used in the VPA animal model of autism (Bambini-Junior et al., 2014; Chaliha et al., 2020; Mabunga et al., 2015; Ross et al., 2015; Schneider & Przewłocki, 2005). The data presented in this study are novel since, to our knowledge, no study in the current literature has compared rats exposed to these two compounds at two points of development such as adolescence and adulthood.

Considering the possibility of the prenatal effects of CPF and VPA during development, the animals were weighed during their first stage of development, and no alteration was found. In addition, the expression of AChE activity was measured in these groups, and no significant differences were found between them (Morales-Navas et al., 2020).

For adolescent behavior, in Phase 1, we have found that the CPFs show less locomotor activity than the rest of the groups. Although the opposite it was expected due to the comorbidity between ADHD and ASD is high (Antshel et al., 2013; Sokolova et al., 2017). However, ASD appears to have a very complex etiology, with many different factors, so this comorbidity is not total.

Concerning the sociability shown by the adolescent subjects (Phase 2), although there are within-group differences, we did not find any significant differences between groups using the sociability index. This result in VPA is in line with another investigation that used a dose identical to ours (Bambini-Junior et al., 2014). Thus, they are showing a behavior within our hypothesis expectations.

In Phase 3, relating to the reaction to the social novelty of adolescents, we observed clearly how CPFs and VPAs behave almost identically in comparison to CNTs. This is further supported by the difference we found in the reaction to social novelty indexes. More specifically, for the perimeter of the entire habitat (SNI), the CPF rats differed significantly from the CNT group but not the VPAs, which appear to be very similar to the CPFs. This data obtained with VPA has been observed in other studies, such as that of Kim et al. (2014), in which an equivalent dose was used. Other similar experiments using CPFs, such as that of Venerosi et al. (2008) or Perez-Fernandez and et al. (2020a, b), did not show any difference in this phase, possibly because none of these studies used prenatal doses.

Turning to the results found for adults with the 3-chambered paradigm, a somewhat different picture emerges. In Phase 1, the differences between the CPF and the other two groups completely disappear, which may be directly due to the passage of time since it is known that adolescent rats are more active (Spear, 2000).

In the social phase, CNTs and CPFs exhibited similar behavior, while VPAs didn't show a clear preference for Stranger 1. These findings are supported by the sociability indexes, both in the SI and the SNI-Contact (the latter shows stability in the data). The difference was between the CPFs and the VPAs, with the CNTs remaining as an intermediate group. This change could be because the systemic damage caused by VPA could be more severe and sustained over time compared to that caused by CPF. It is important to remember that VPA is a widely accepted model of autism in the scientific community, while the CPF has received more criticism in this regard (Williams & Desesso, 2014).

In the reaction to the social novelty phase, CPFs and VPAs displayed a preference for the new conspecific, something that surprisingly did not occur with the CNT group, which was expected to spend more time with Stranger 2 (Crawley, 2004; Moy et al., 2004). What could be happening in this case is a bidirectional effect. While VPA-exposed animals benefit from being raised alongside non-autistic animals and environmental enrichment (Campolongo et al., 2018; Schneider et al., 2006; Yamaguchi et al., 2017), CNT animals are also influenced by the VPAs. In between would be the CPF animals, representing a much “weaker” version of certain autistic characteristics, and therefore, subject to a more noticeable improvement in the social aspects. However, this possibility is merely speculative and should be tested with further research.

On the basis of this study, it seems that animals prenatally exposed to subclinical doses of CPF at days 12.5–15.5 show certain social characteristics similar to those observed in animals exposed to VPA, which shifts from a decline in adolescence (this alteration being more pronounced than that shown by VPA animals), to a recovery in adulthood. This finding could be due to co-breeding with CNT congeners and environmental enrichment. The stability found in VPA animals—the positive control model—could mean that there are more difficulties in recovering them by mere upbringing or a later life shared with other congeners who do not present the same disorder.

Thus, given all the above, and with the knowledge that more research is needed, CPF is a compound that should be considered as a risk factor in the development (either as an aggravator or elicitor) of certain dysfunctions related to the etiology of ASD.

## Supplementary Information

Below is the link to the electronic supplementary material.

Supplementary file1 (DOCX 2525 kb)
