# Supplementary material for: Sociability: Comparing the Effect of Chlorpyrifos with Valproic Acid
Source: J Autism Dev Disord. 2024 Mar 11;55(3):1101–11. doi: 10.1007/s10803-024-06263-z (PMC11828833; doi:10.1007/s10803-024-06263-z)
Supplement: Supplementary file 1 — Supplementary file1 (DOCX 2525 kb) [file 10803_2024_6263_MOESM1_ESM.docx]

**APPENDIX**


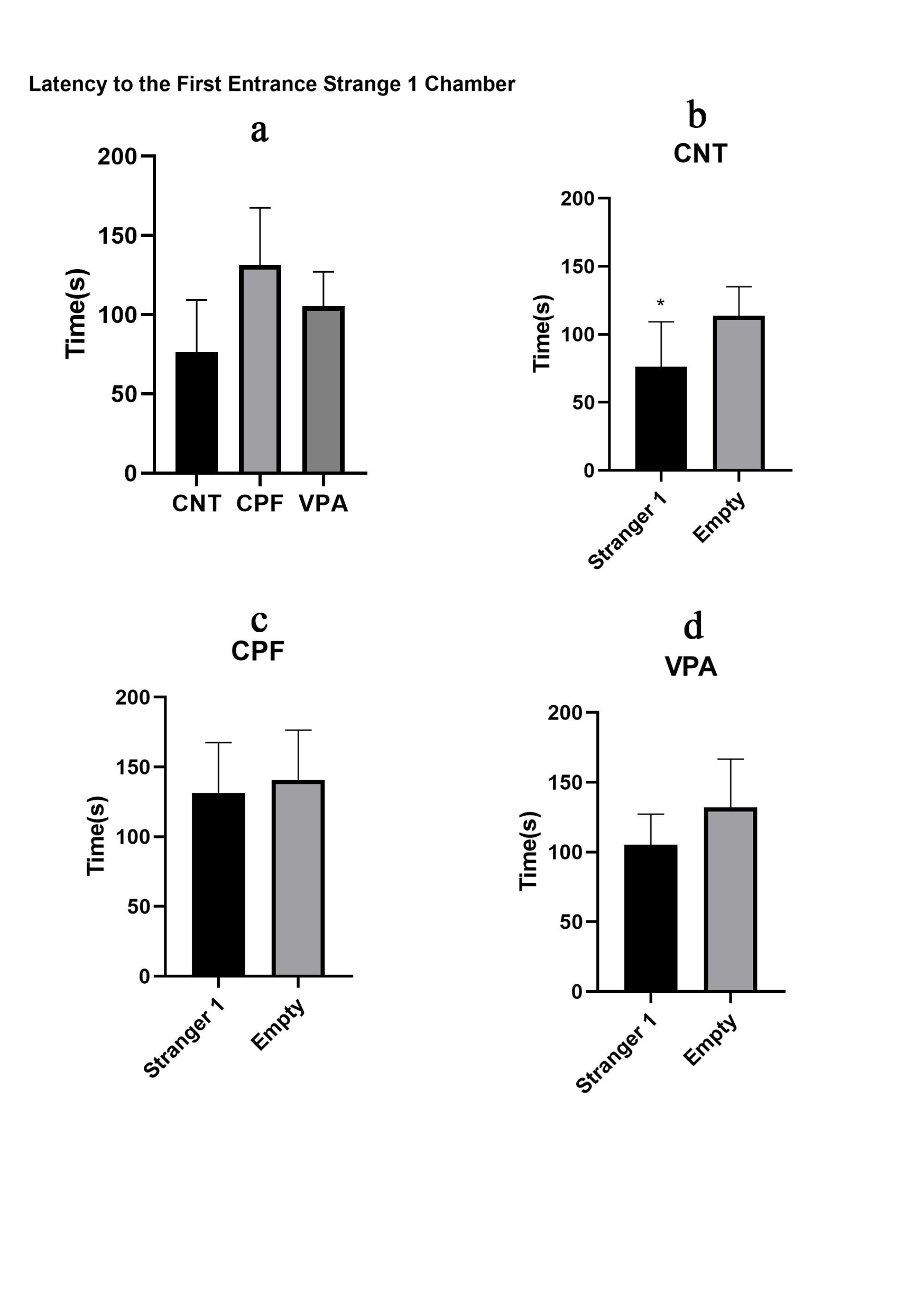


**Fig.11** Latency of first entry into the Stranger 1 chamber compared with entry into the Familiar chamber, in adolescents - Phase 2 (n = 61; CNT = 20 [10 females and 10 males], CPF = 21 [11 females and 10 males], VPA = 20 [10 females and 10 males]): In the time elapsed before first entry into the Stranger 1 chamber, no significant differences were observed between groups (a). However, within groups, CNTs (b) take significantly less time to enter the Stranger 1 chamber than the other two groups (c and d)


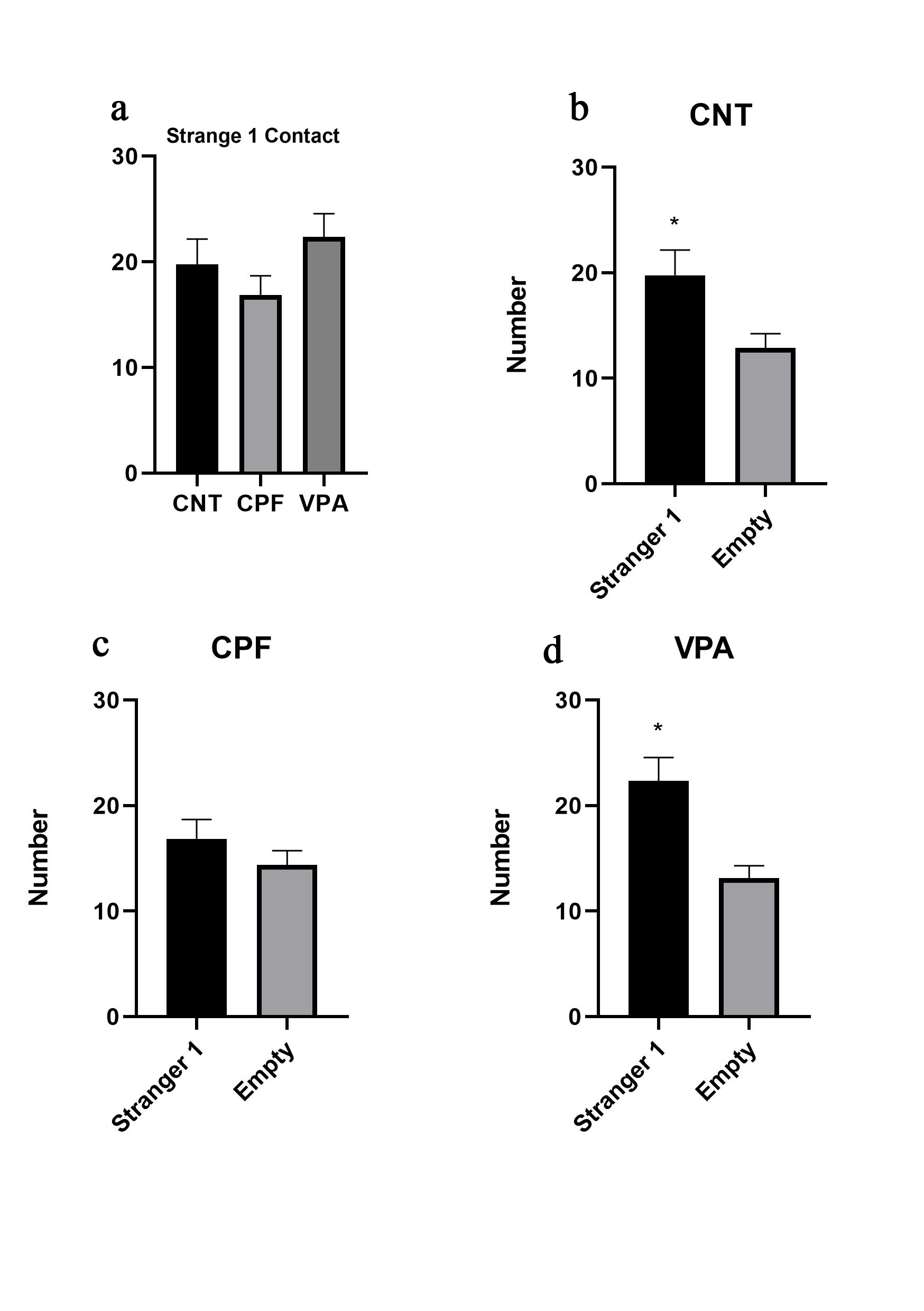


**Fig.12** Frequency of entries in contact with Stranger 1 compared with those of the Familiar chamber, in adolescents - Phase 2 (n = 61; CNT = 20 [10 females and 10 males], CPF = 21 [11 females and 10 males], VPA = 20 [10 females and 10 males]): In the number of entries, no significant differences were found between the different treatments (a). However, within the groups, we observe how CNTs and VPAs enter the chamber significantly more times (b and d, respectively), while this difference does not exist in the CPFs (c)


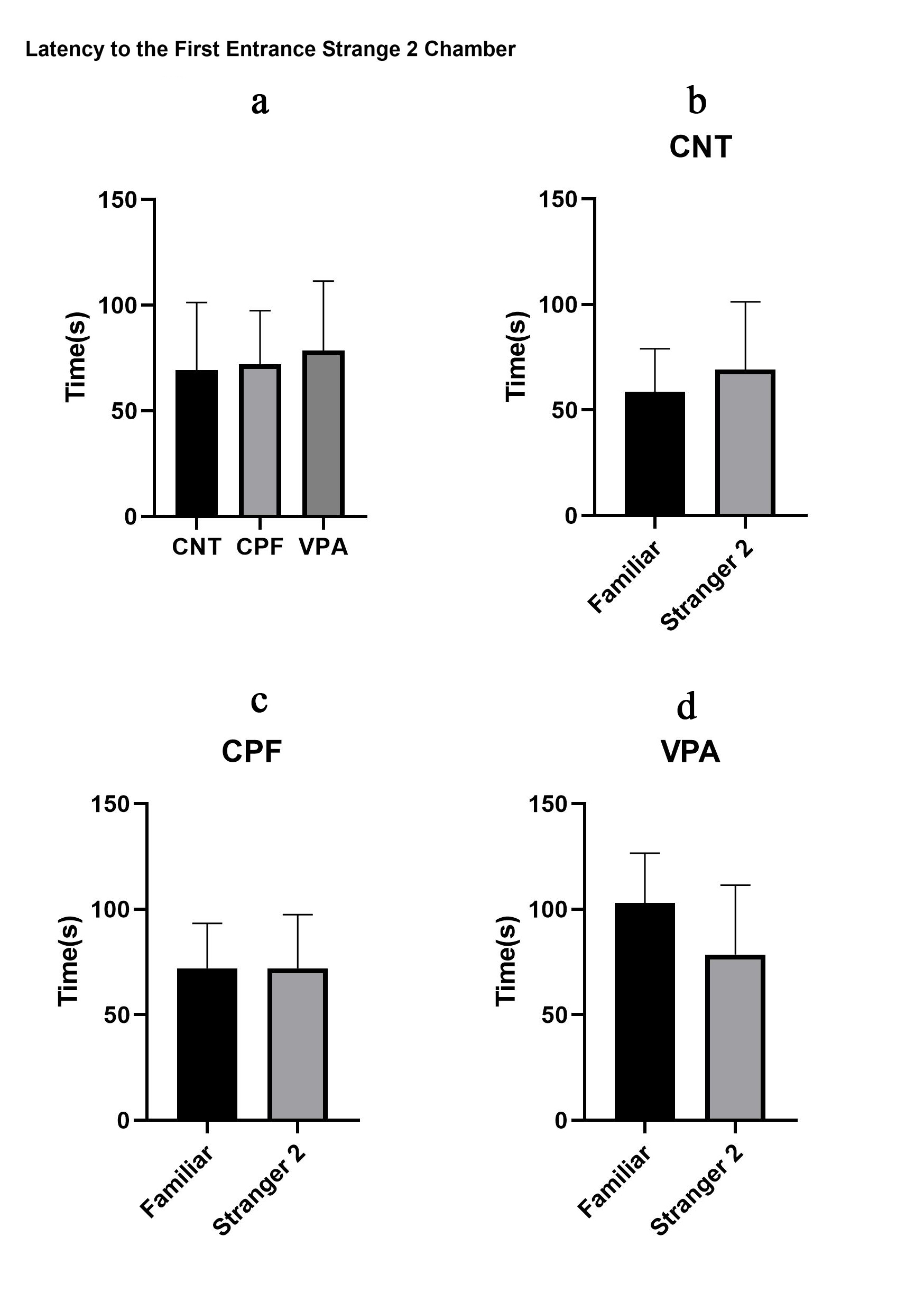


**Fig.13** First entry latency in the Stranger 2 chamber, as well as in comparison with that in the Relative chamber, in adolescents - Phase 3 (n = 54; CNT = 16 [10 females and six males], CPF = 19 [10 females and nine males], VPA = 19 [9 females and ten males]): The different experimental groups show no significant differences concerning the time taken to approach the new conspecific (Stranger 2) (a). Likewise, there are no differences between the time it takes to see the Familiar or Stranger 2 in any of the groups separately (b, c, and d)


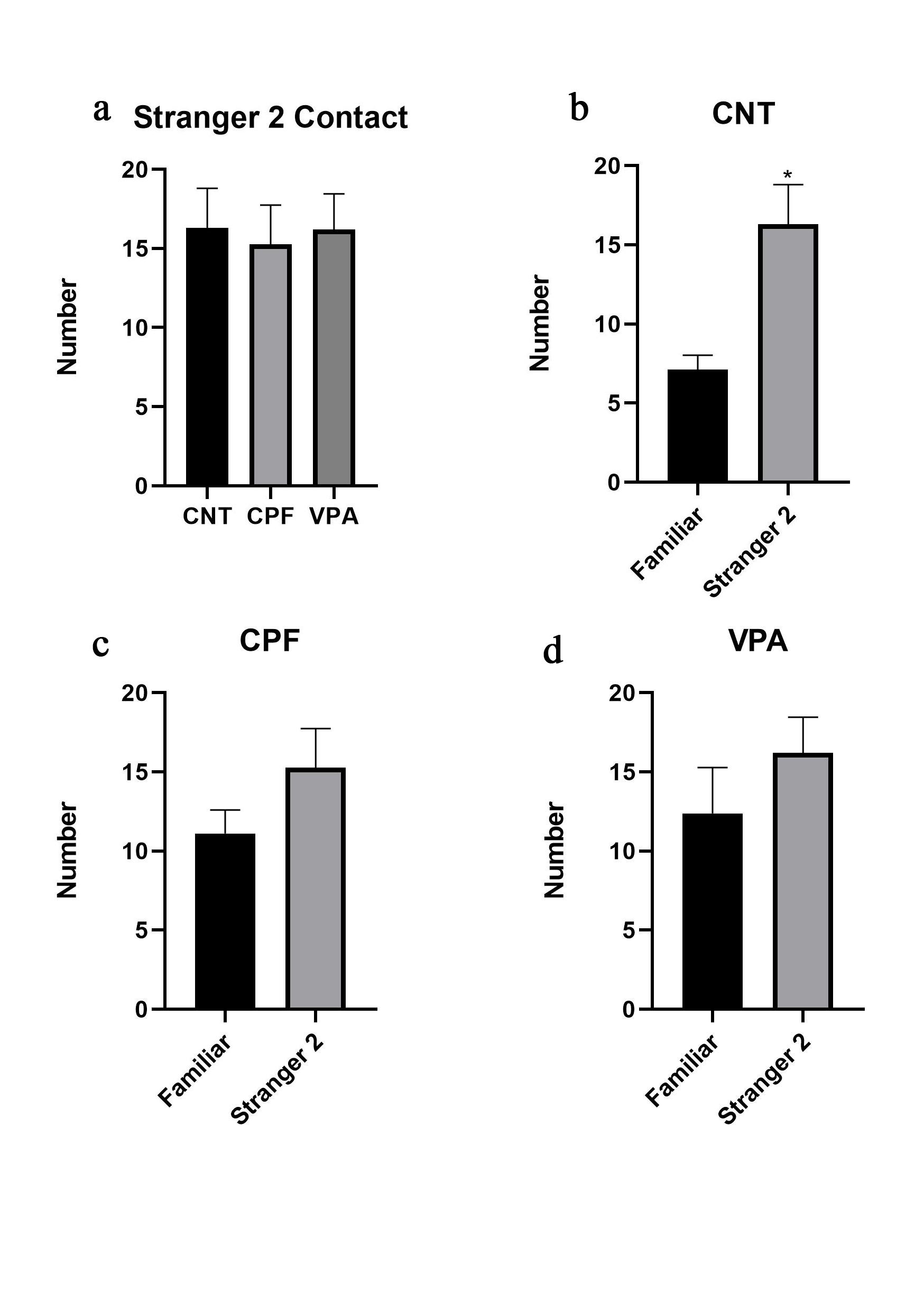


**Fig.14** Frequency of entries in contact with Stranger 2, as well as in comparison with those in the Familiar chamber, in adolescents - Phase 3 (n = 54; CNT = 16 [10 females and six males], CPF = 19 [10 females and nine males], VPA = 19 [9 females and ten males]): The different experimental groups do not show significant differences in the times they enter the Stranger 2 (a) habitat. However, CNTs show significant differences in the times they enter the contact perimeter with Stranger 2 (b). While CPFs and VPAs do not show significant differences between the entries, they make into the habitat where the Familiar is located concerning Stranger 2 (c and d, respectively)


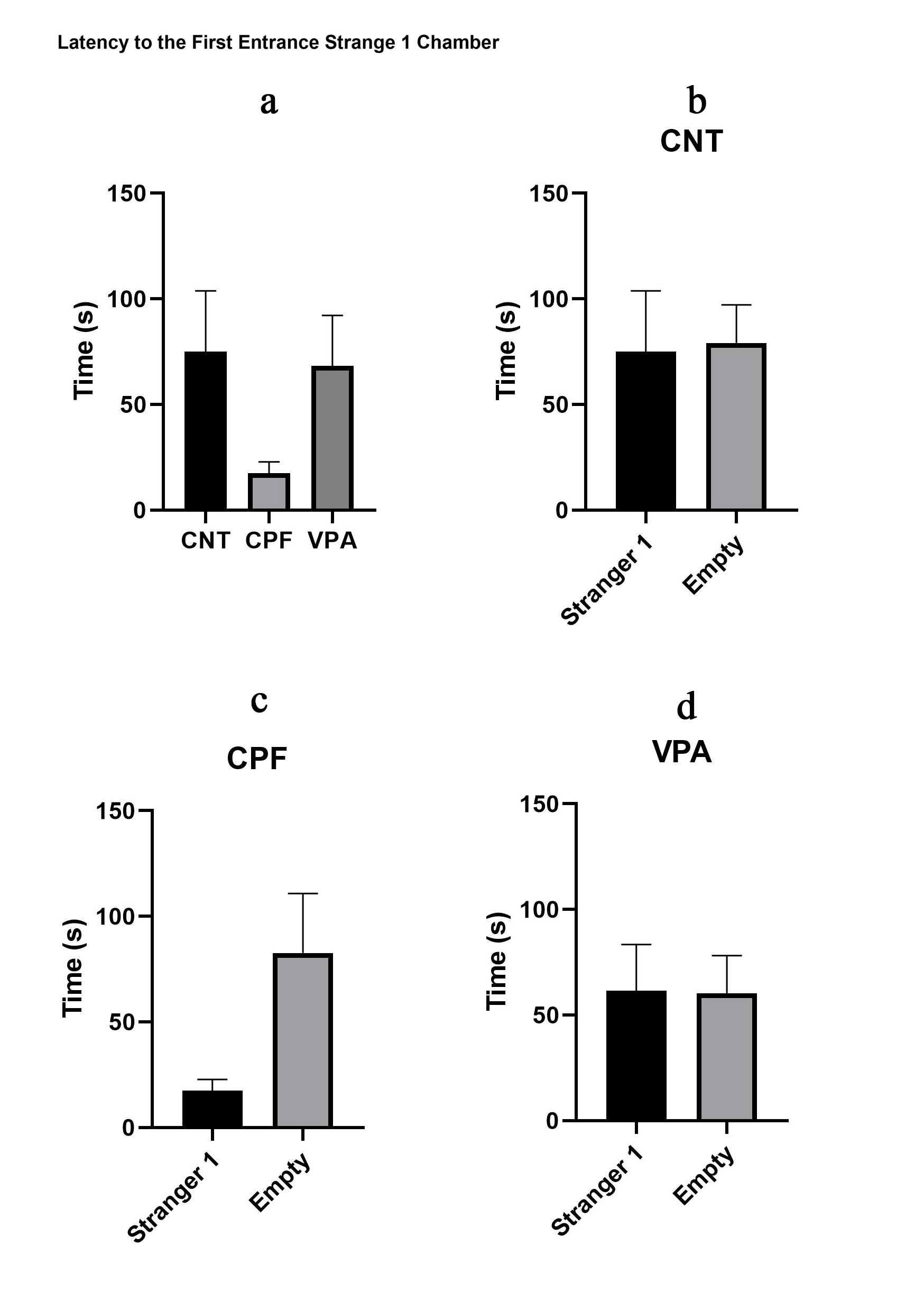


**Fig.15** First entry latency in the Stranger 1 chamber, as well as in comparison with that in the Familiar chamber, in adults - Phase 2 (n = 61; CNT = 20 [10 females and 10 males], CPF = 21 [10 females and 11 males], VPA = 20 [10 females and 10 males]): Although there is a strong tendency for CPFs to take less time to visit Stranger 1 compared to the other treatments, there are no significant differences (a). Furthermore, although this tendency is also visualized in the comparative analyses of each group and the two chambers: Stranger 1 and the empty chamber, there are no significant differences in any of the groups separately (b, c, and d)


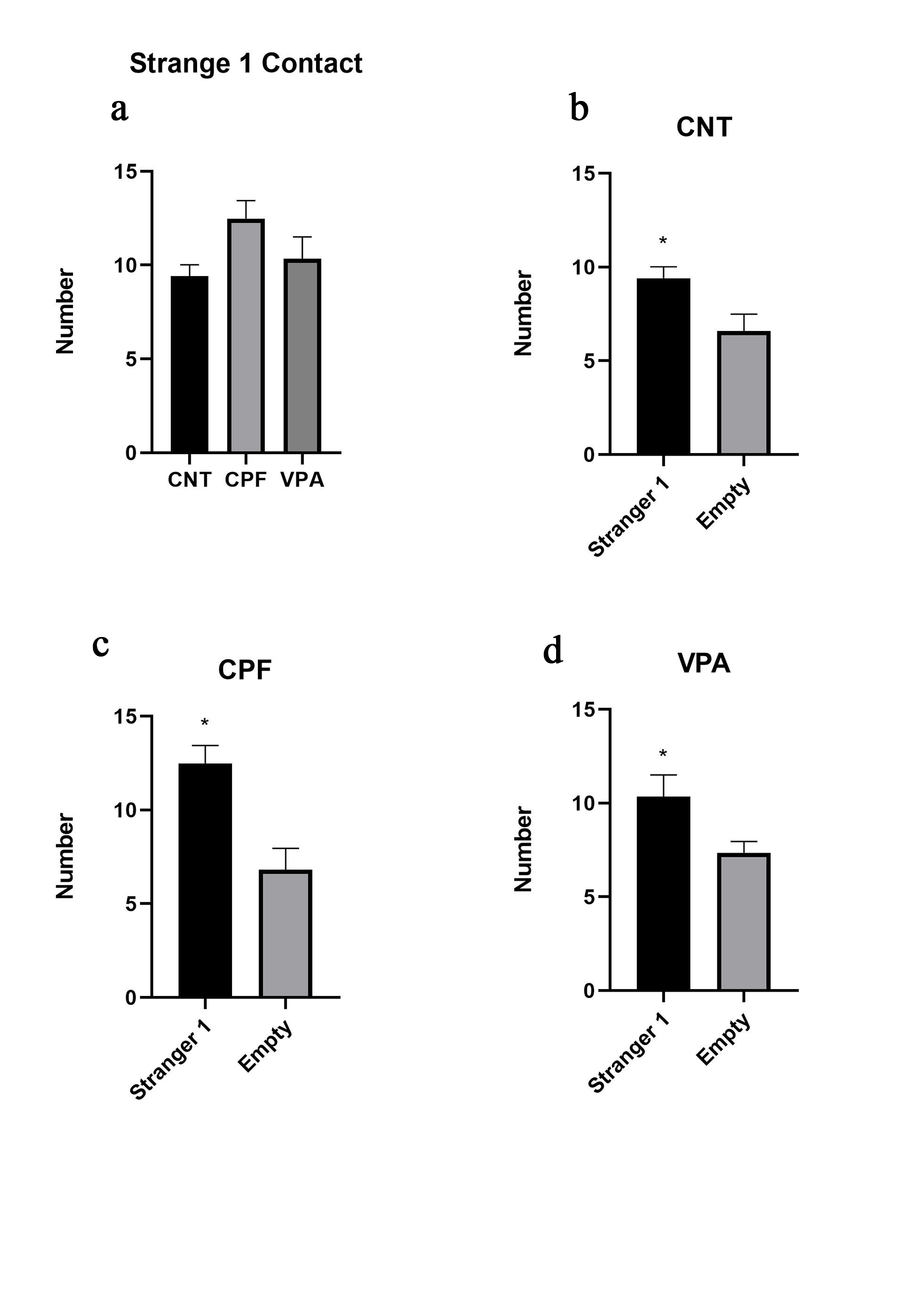


**Fig.16** Frequency of entries in contact with Stranger 1, as well as in comparison with those in the Familiar chamber, in adults - Phase 2 (n = 61; CNT = 20 [10 females and 10 males], CPF = 21 [11 females and 10 males], VPA = 20 [10 females and 10 males]): No significant differences are observed between treatments (a). However, all groups make more entries to the Stranger 1 contact zone than where the empty cage is located (b, c, and d, respectively)


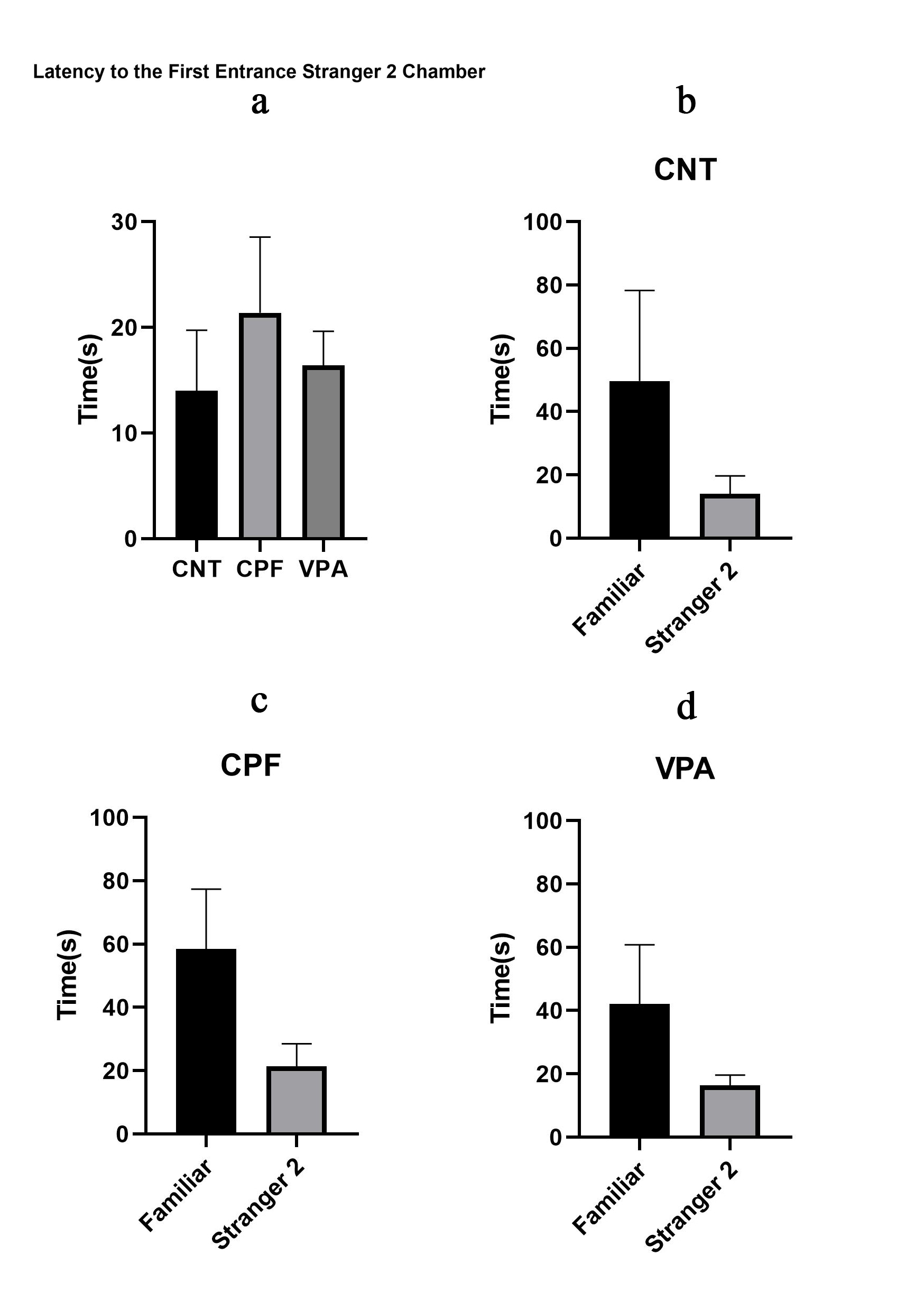


**Fig.17** First-entry latency in the Stranger 2 chamber in the adult 3-bedroom test - Phase 3 (n = 50; CNT = 15 [9 females and 6 males], CPF = 18 [9 females and 9 males], VPA = 17 [9 females and 8 males]): Between groups, we found no significant differences (a). Nor did we find any significant differences in the CNTs (b), CPFs (c), or VPAs (d)


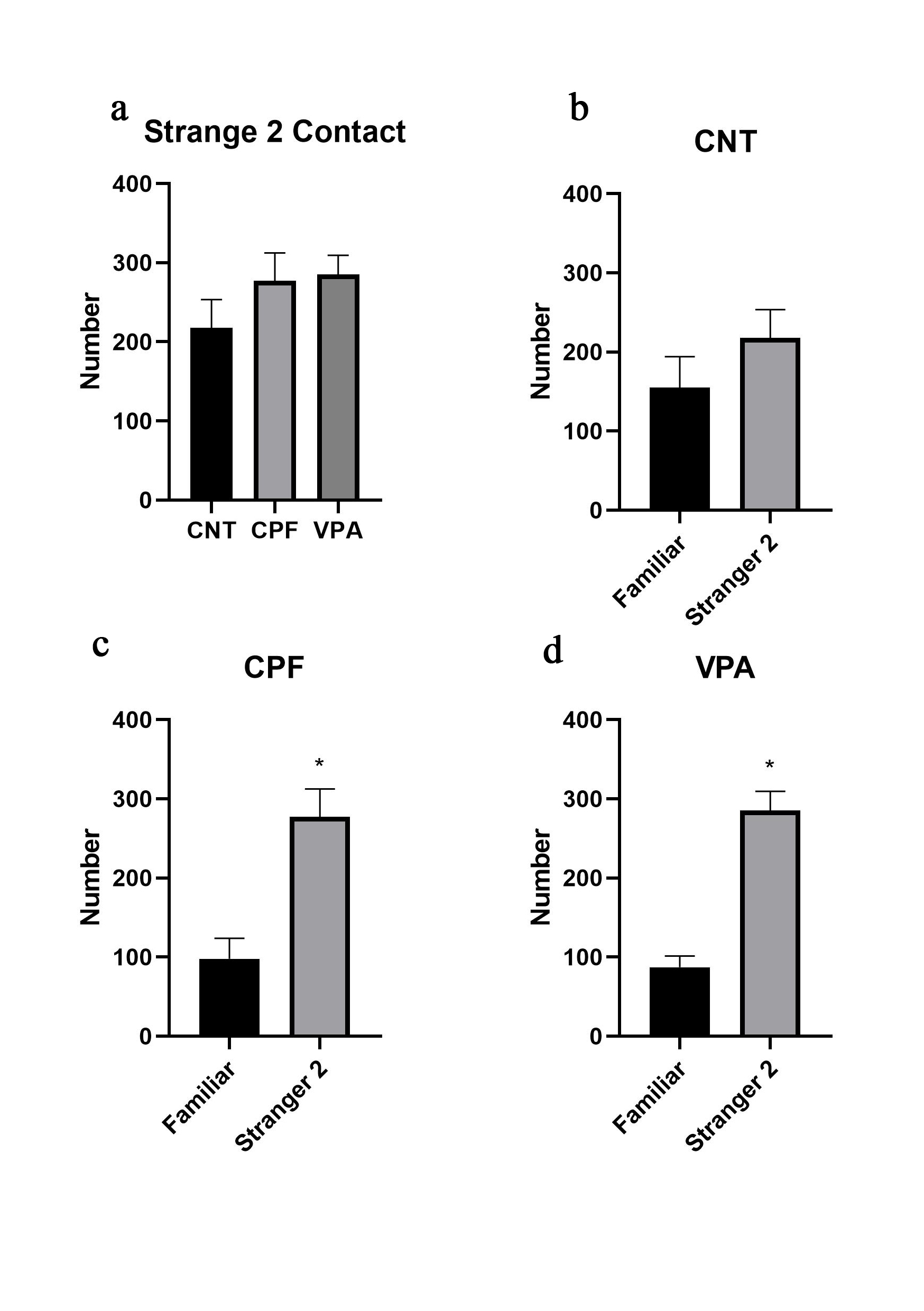


**Fig.18** Number of entries in the Stranger 2 chamber - Phase 3 n = 50; CNT = 15 [9 females and 6 males], CPF = 18 [9 females and 9 males], VPA = 17 [9 females and 8 males]): No significant differences are observed between treatments (a), nor in the intrinsic comparison of CNTs (b). On the contrary, we observe how CPFs and VPAs make more entries in contact with Stranger 2 (c and d, respectively)
